# Lameness Detection in Dairy Cows: Part 1. How to Distinguish between Non-Lame and Lame Cows Based on Differences in Locomotion or Behavior

**DOI:** 10.3390/ani5030387

**Published:** 2015-08-28

**Authors:** Annelies Van Nuffel, Ingrid Zwertvaegher, Liesbet Pluym, Stephanie Van Weyenberg, Vivi M. Thorup, Matti Pastell, Bart Sonck, Wouter Saeys

**Affiliations:** 1 Technology and Food Science Unit–Precision Livestock Farming; The Institute for Agricultural and Fisheries Research (ILVO), Burgemeester van Gansberghelaan 115 bus 1, 9820 Merelbeke, Belgium; E-Mails: ingrid.zwertvaegher@ilvo.vlaanderen.be (I.Z.); liesbet.pluym@ilvo.vlaanderen.be (L.P.); stephanie.vanweyenberg@ilvo.vlaanderen.be (S.V.W.); 2 INRA, UMR 791 Systemic Modelling of Ruminant Nutrition, 16 rue Claude Bernard, 75231 Paris cedex 05, France; E-Mail: vivi.thorup@agroparistech.fr; 3 AgroParisTech, UMR 791 Systemic Modelling of Ruminant Nutrition, 16 rue Claude Bernard, 75231 Paris cedex 05, France; 4 Natural Resources Institute Finland (Luke), Green Technology, Koetilantie 5, 00790 Helsinki, Finland; E-Mail: Matti.pastell@luke.fi; 5 Animal Sciences Unit, The Institute for Agricultural and Fisheries Research (ILVO), Scheldeweg 68, 9090 Melle, Belgium; E-Mail: bart.sonck@ilvo.vlaanderen.be; 6 Department of Biosystems Engineering, Faculty of Bioscience Engineering, Ghent University, Coupure links 653, 9000 Gent, Belgium; 7 Division Mechatronics, Biostatistics and Sensors (MeBioS), Department of Biosystems, Katholieke Universiteit Leuven, Kasteelpark Arenberg 30 bus 2456, 3001 Heverlee, Belgium; E-Mail: wouter.saeys@biw.kuleuven.be

**Keywords:** lameness, dairy cattle, cow gait, behavior, visual locomotion scoring

## Abstract

**Simple Summary:**

Scoring cattle for lameness based on changes in locomotion or behavior is essential for farmers to find and treat their lame animals. This review discusses the normal locomotion of cows in order to define abnormal locomotion due to lameness. It furthermore provides an overview of various relevant visual locomotion scoring systems that are currently being used as well as practical considerations when assessing lameness on a commercial farm.

**Abstract:**

Due to its detrimental effect on cow welfare, health and production, lameness in dairy cows has received quite a lot of attention in the last few decades—not only in terms of prevention and treatment of lameness but also in terms of detection, as early treatment might decrease the number of severely lame cows in the herds as well as decrease the direct and indirect costs associated with lameness cases. Generally, lame cows are detected by the herdsman, hoof trimmer or veterinarian based on abnormal locomotion, abnormal behavior or the presence of hoof lesions during routine trimming. In the scientific literature, several guidelines are proposed to detect lame cows based on visual interpretation of the locomotion of individual cows (*i.e.*, locomotion scoring systems). Researchers and the industry have focused on automating such observations to support the farmer in finding the lame cows in their herds, but until now, such automated systems have rarely been used in commercial herds. This review starts with the description of normal locomotion of cows in order to define ‘abnormal’ locomotion caused by lameness. Cow locomotion (gait and posture) and behavioral features that change when a cow becomes lame are described and linked to the existing visual scoring systems. In addition, the lack of information of normal cow gait and a clear description of ‘abnormal’ gait are discussed. Finally, the different set-ups used during locomotion scoring and their influence on the resulting locomotion scores are evaluated.

## 1. Introduction

The increasing demand for animal products has led to a rapid growth in livestock production, including dairy farming, during the last 20 years [1]. As a result, dairy farming systems worldwide have intensified, with more cattle on fewer farms and per caretaker and higher productivity per animal [2]. This trend reduces the farmer’s available time to observe and monitor the cows and jeopardizes the health of the cows, in particular the high-yielding ones. Lameness is considered to be the third most costly health problem of dairy cows, after reduced fertility and mastitis [3]. Nevertheless, lameness has not only been under-recorded on farms but its importance with regard to cow welfare, cow health and farm profitability has also been hugely underestimated [4], even though it induces both direct (drug treatment, veterinary costs and death) and indirect costs (reduced milk production, reproductive performance and life expectancy) [5]. With a lameness prevalence reaching up to 72% [6], the levels in dairy herds in Europe are unacceptably high. Therefore, minimizing the occurrence and impact of lameness is one of the greatest challenges the dairy industry is currently facing [7]. Unfortunately, many dairy farmers are unaware of the number of lame cows in their herd, and, if noticed, they often do not have enough time to treat them [8]. Because many farmers fail to notice or lack the time to look for lame cows, sensor technology is being used to develop automated lameness detection systems. Generally, lame cows are detected by the herdsman, hoof trimmer or veterinarian based on changes in cow gait, posture or behavior or the presence of hoof lesions during routine trimming. This review is the first of two focusing on cow characteristics that are used to visually detect lame cows in practice. These characteristics are grouped into those of cow gait, posture and general behavior. To know which alterations of these characteristics are due to lameness, information on the normal locomotion (gait and posture) and behavior of cows are firstly described. Next, the abnormal characteristics in cow gait, posture or behavior closely related to lameness are summarized. In addition, several lameness scoring systems are compared, and some considerations in applying them in practice are discussed. The second review focuses on sensor technology to automatically measure relevant cow characteristics of gait, posture or behavior to develop automated lameness detection systems that support farmers in identifying the lame cows in their herd [9]. 

## 2. Definition of Lameness

Lameness can be defined as the clinical manifestation of painful disorders, mainly related to the locomotor system, resulting in impaired movement or deviation from normal gait or posture. The severity of lameness can vary from stiffness or decreased symmetry of limb movement to an inability to bear weight on a limb, or even total recumbency [10,11].

Disorders can be located in the limbs or trunk of the animal, and may include painful lesions as well as mechanical defects resulting in physical incapability [12]. Gait problems are thus a manifestation of discomfort or pain. In dairy cattle, the main cause of lameness are claw lesions. Claw lesions can be divided into non-infectious (such as white line disease, sole ulcer, sole hemorhhage, interdigital hyperplasia) or infectious claw lesions (such as digital dermatitis, interdigital dermatitis, heel erosion, foot rot) [13]. In addition, lameness can also be associated with injury in the nervous system (such as obturator paralysis) as well as the musculoskeletal system (such as fractures, arthritis and tendonitis) [14]. 

How these different disorders specifically influence the gait of cows remains unknown. However, to recognize the region of the hoof in which the cause of the lameness is located, the Merck Veterinary Manual [14] suggested the following: “when there is pain in the toe, the retraction phase (when hoof passes behind the phase of vertical weight-bearing) of the stride is reduced considerably. In contrast, if the pain is located in the heel, the protraction phase of the stride is reduced or the hoof is not carried as far forward as is normal”. To define whether the cause of lameness is in the hoof or the upper limb, Jackson and Cockcroft [15] stated that supporting limb lameness (*i.e.*, shortened weight bearing and quick swing phase in order to minimize the contact with the ground) is associated with lesions in the hoof whereas swinging leg lameness (limb is held in extension during wing phase as flexion causes pain) with disorders in the upper limb. 

Yet, it should be noted that abnormal gait may develop not only as a result of disorders in the locomotor system but also disorders in other organs (e.g., severe udder distension in heifers) [16]. Furthermore, both environmental and cow factors may impair locomotion. Wet flooring, dark environments, time relative to claw trimming sessions, age, cow dimensions, lactation and gestation stage might cause normal changes in cow gait that are not related to lameness [17]. 

In this review, lameness is considered abnormal locomotion due to pain caused by the locomotor system. Although changes in the general behavior of cows, like lying, standing or feeding behavior, have been associated with lameness, changes in locomotion are the most commonly used and most direct ways to monitor lameness. 

## 3. Normal Locomotion of Dairy Cows

To understand abnormal locomotion (*i.e.*, gait and posture), thorough insight into normal gait—with an emphasis on the way cows walk—is essential. Fundamental gait analyses have focused on the relation between limb/hoof events, which are traditionally described in terms of footfall patterns or phase relationships between limbs [18]. 

Phillips [19] defined walking as strides where each limb is lifted by shortening of the limb through flexion of the joints using especially hip, knee, hock and digital flexor muscles. The limb then enters the swing phase (during which the limb has no contact with the ground) and is placed on the ground through slow extension of the joint. Once the limb is on the ground, it checks the support and the sole is pushed hard against the floor by contracting the digital flexors. This enables the start for the support phase (during which the limb is in contact with the ground) again followed by the next swing phase. 

The walk is a gait of four evenly spaced beats with no suspension phase (*i.e.*, the phase in which the animal moves forward without any limb touching the ground) but with alternate support by two or three limbs. The usual succession of ground-contacting limbs during walk is LH–LF–RH–RF (where L, R, H, F indicate left, right, hind, and front, respectively) with a regular rhythm and even spacing between footfalls. Hildebrand [20] stated that it is conventional to enter the cycle with the footfall of the left hind foot. To allow the hind limb to overlap the front limb imprint, the latter is usually lifted just before the hind limb is placed. During a normal walk, the duration of support exceeds that of swing, and different limbs show less than 50% overlap in the swing phase of two successive steps [18]. 

**Figure 1:**
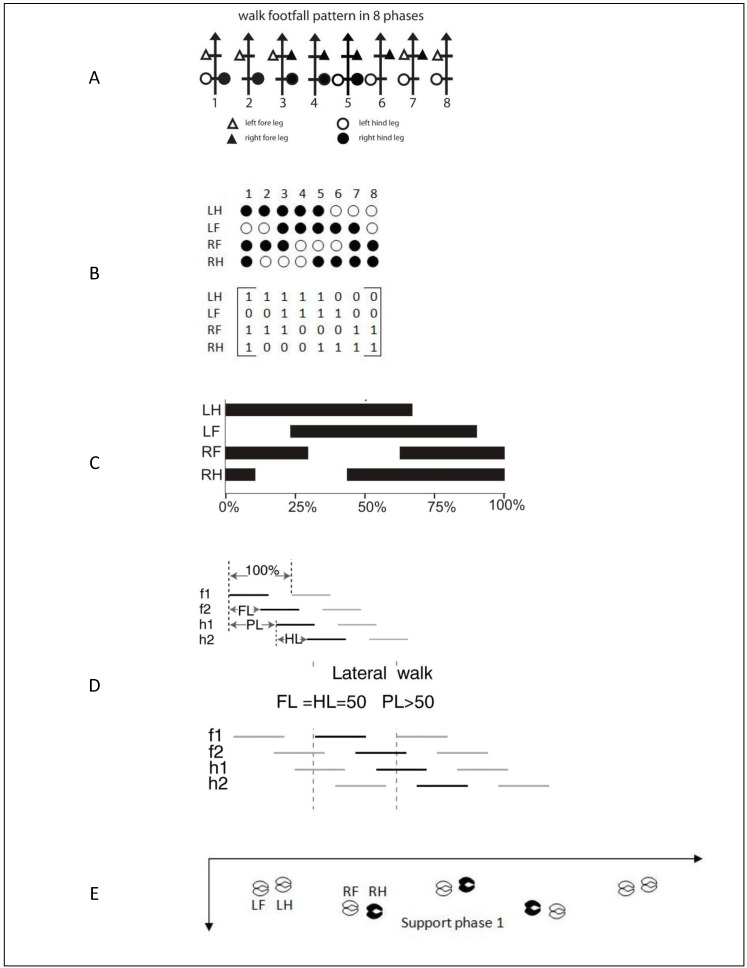
Schematic representation of quadruped gait: (**A**) footfall sequence formula according to Muybridge [21] with compact notation according to Hildebrand [20]; (**B**) gait matrix [22]; (**C**) gait diagram of a common lateral walk with additional temporal information [20]; (**D**) track-way diagrams revealing the use of the F-, H- and P-lag of Abourachid [23]; (**E**) spatial distribution of the supporting limbs [24].

Several techniques have been proposed to schematically represent animal gait, resulting in different ways to describe gait. Muybridge [21] proposed the footfall sequence formula (Figure 1A), while McGhee [22] defined the equivalent gait matrix (Figure 1B). Hildebrand’s [20] representation of the relative duration of the stance and swing phases during one stride are illustrated in Figure 1C. Abourachid [23] introduced an antero-posterior sequence approach in analysing gaits of quadrupeds where—besides focusing on strides—the time lags between the movements of both front limbs (F-lag) or hind limbs (H-lag) or between the front and hind limbs of the same side (P-lag) were added (Figure 1D). If the percentage of time lag during the whole cycle is 50% for both F-lag and H-lag and > 50% for P-lag, the walk is called lateral. Telezhenko [24] proposed track-way diagrams to describe the spatial relation between different limbs (Figure 1E).

**Figure 2:**
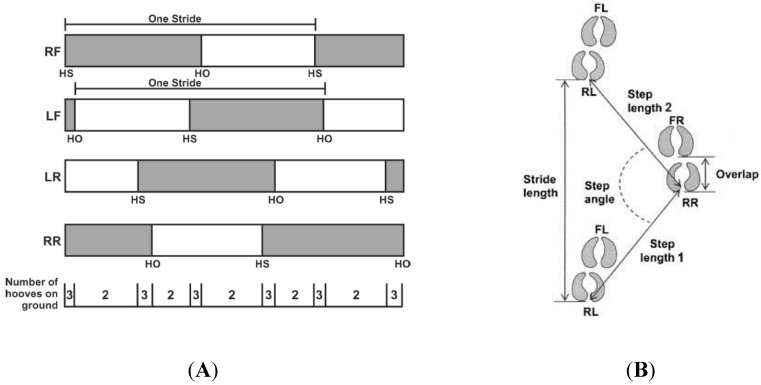
Temporal (**A**) and spatial (**B**) representation of cow gait: (**A**) the mean proportion of double and triple support time during a single stride for each limb; RF = right front hoof, LF = left front hoof, LR = left rear hoof, RR = right rear hoof, hoof strike (HS), and hoof-off (HO) [25]; (**B**) spatial track way measurements as performed by Telezhenko [24]; FL = front left; RL = rear left; FR = front right, and RR = rear right.

Detailed information on footfall patterns in Holstein dairy cows was provided by Flower *et al*. [25] and Telezhenko [24] using a temporal gait diagram and a track-way representation of the spatial distribution of all limbs, respectively. Flower *et al*. [25] found that for non-lame cows only 18% of the stride time was in triple support, *i.e.*, time when the body weight is supported by three limbs instead of two (Figure 2A). Telezhenko [24] reported three types of possible track-way measurements that either describe the rate of progression (stride length and tracking), the balance (step width, step angle) or the coordination between different limbs (step length, step asymmetry). Some of these variables are visualized in Figure 2B. 

Besides spatio-temporal characteristics of gait, the forces exerted by the claws on the ground have been measured. One of the first pressure measurements in cattle was performed by Distl and Mair [26]. They reported an average force of 19 N/cm^2^ with peaks of 56 and 59 N/cm^2^ for first and second parity cows, respectively. Rajkondawar *et al*. [27] measured peak and averaged ground reaction forces (relative to the cow’s body weight) resulting in dimensionless numbers of 0.41 N/N and 0.30 N/N, respectively, and a stance time of 1.29 s for the hind limbs during walk. The majority of the total body weight (54% to 60%) is supported by the front limbs [19,28,29].

**Table 1:** Average (±stdev) values of gait variables for non-lame cows.

Gait Variable	Average ± stdev
Walking speed (m/s)	1.350 ± 0.150 ^1^
Stride length (m)	1.591 ± 0.005 ^2^
Stride time (s)	1.523 ± 0.009 ^2^
Stance time (s)	1.011 ± 0.007 ^2^
Step overlap (m)	0.011 ± 0.003 ^2^
Abduction (m)	0.029 ± 0.001 ^2^
Asymmetry in step width (m)	0.201 ± 0.003 ^2^
Asymmetry in step length (m)	0.419 ± 0.002 ^2^
Asymmetry in step time (s)	0.389 ± 0.002 ^2^
Asymmetry in stance time (s)	0.024 ± 0.001 ^2^
Leg weight ratio (/) *	80.0 ^3^

^1^ Thorup *et al*. [30], n = 348 cows; ^2^ Van Nuffel [17] n = 34 cows; ^3^ Pastell and Kujala [31], n = 73 cows; * Leg weight ratio is the ratio between the heavier and lighter hind leg.

Mean values of locomotion characteristics of non-lame cows are provided in Table 1. Differences in gait variable measurements reported in the literature might be explained by differences in the breed, cow dimensions and cow factors (parity, locomotion scores, lactation stage, *etc*.) of the animals in the study, the measurement techniques used, measurement set-up or the definitions of the variable. Telezhenko [24] indeed found significant correlations (R^2^ of 0.51–0.66) between the height and the length of the cow and stride and step length, step angle and width. In a pilot study by Van Nuffel [17], step length and speed for example decreased with age and gestation stage. However, no information on cow dimensions (height, length, breast width, udder size, *etc*.) was collected during this study. 

The weight of the cow is carried by the claw. The claw wall bears most of the load during walking, while during standing, most pressure was found at the heel area of the claw [32,33,34]. In a more detailed study done by van der Tol *et al*. [35], up to 51% (front limbs) and 37% (hind limbs) of the total body weight of the cows is almost completely borne by the lateral part of the claw compared to the medial part during walk, especially at heel strike. When standing, the posterior part and anterior of the claw are most often subjected to the highest pressure in the front limbs and hind limbs, [36]. Hence, depending on the lesion type, severity and location, a direct association can or cannot be found between the weight applied to the limb and the lesions present [37,38]. Moreover, when cows are lame on both hind limbs, weight is seldom transferred to the front limbs in an attempt to reduce pressure on the painful limbs. In contrast, cows that were lame on both front limbs were found to transfer some of the weight to the hind limbs [28]). However, the fact that painful lesions often occur symmetrically makes detection of lameness even more difficult.

Body movement patterns of the head, spine, joints and tail can also be considered characteristics of locomotion [20], yet recent studies of locomotion have mainly focused on the timing and duration of footfalls on the ground and the different patterns of support and swing phases (*i.e.*, gait) and less on posture. 

## 4. Signs of Lameness

### 4.1. Changes in Gait Patterns

In an attempt to detect abnormal or lame gait, several gait characteristics are used in the locomotion scoring of cattle. However, very few papers describe the relationship between poor locomotion and the gait characteristics. Flower *et al*. [16,25] found that lame cows walked slower, had longer stride durations, shorter strides and a more uneven weight distribution over the limbs than non-lame cows. The triple support time in the gait cycle even doubled for lame cows compared with healthy ones. Comparably, Telezhenko *et al*. [39] reported that severely lame cows walked more slowly with shorter stride and step length and a smaller step angle (see Figure 2B). Maertens *et al*. [40] and Blackie *et al*. [41] also found shorter stride lengths and negative tracking distances in lame cows compared with non-lame cows. Tracking distance (also reported as tracking, tracking up or step overlap) can be defined as over- or under-extension of the stride of a hind limb resulting in the hind claw not being placed on the same location as the front claw after initiation of the stride [40,42]. Similarly, increased abduction, meaning the sideways distance between the front foot imprint and the next placement of the hind foot on the same side, has been suggested to be associated with lameness [24,40]. According to Maertens *et al*. [40] lame cows showed more asymmetry between left and right limbs in step width, step length, step time, stance time and relative force compared with non-lame cows. Studies measuring stride heights or stiff movement of the joints have been scarce. Nevertheless, such variables of general flexion of the joints have also been suggested to be related to lameness [25,43]. Van Nuffel *et al*. [44,45] introduced the inconsistency of gait as a possible indicator for lameness based on the hypothesis that cows first occasionally show, e.g., short strides interchanged with ‘normal’ stride lengths before taking shorter strides in general when the lameness becomes more severe.

### 4.2. Changes in Posture or Body Movement Patterns

According to Sprecher *et al*. [46] and Flower *et al*. [16,25], a more pronounced arched-back posture is associated with lameness in cattle, both while standing and walking. Head movements or head ‘bobs’ (*i.e.*, nodding, vertical movements of the head as the lame limb makes contact with the ground) have also been mentioned as a lameness characteristic in cattle [16,25,47]. 

### 4.3. Changes in Weight Distribution Patterns

Lame animals tend to shift their body weight onto non-affected limbs to reduce pain [48]. Indeed, when ground reaction forces were measured for cows during walking, the average and peak ground force reactions were found to decrease with an increase in locomotion score. This suggests that these forces could be used to discriminate lame from non-lame cows [27,49,50]. 

In practice, measuring weight distributions between the limbs of cows is more feasible during stance. Not surprisingly, lame cows placed more weight on the healthy limb in comparison to healthy animals which distributed their body weight more evenly [31]. According to Rushen *et al*. [48], lame cows had more than the normal amount of weight on the limb that was contralateral to the injured limb. The more severe the degree of lameness, the clearer the relationship with the body weight distribution was. However, painful lesions often occur symmetrically, making detection of lameness even more difficult. When cows are lame on both hind limbs, weight is seldom transferred to the front limbs in an attempt to reduce pressure on the painful limbs [28]. In contrast, cows that were lame on both front limbs were found to transfer some of the weight to the hind limbs [28]. 

These results were confirmed by measuring weight shifting between the contralateral limbs [28,37,38,51,52]. Higher leg weight ratios and higher variations in weight shifts between limbs were successfully used to distinguish lame from non-lame cows [31]. 

Increased step and kick behavior during milking has also been highlighted as a good indicator for lameness [51,53,54]. Chapinal and Tucker [55] confirmed these findings. In their study, lame cows stepped more with the hind limbs compared with non-lame cows, although this was not the case for the front limbs. The number of steps was found to be closely correlated to the weight shifting between limbs during measurements and with the limb weight ratio. However, in the study by Rousing *et al*. [56] a higher kicking frequency during milking was associated with pain and discomfort related to teat injuries, whereas no relation between lameness and kicking or stepping behavior was found.

### 4.4. Changes in Behavior 

Besides locomotion, the most important natural behaviors for cow health, welfare and productivity are resting, eating, ruminating, and socializing [57]. Lameness has been associated with longer lying times, longer periods of standing in alleys and decreased feeding behavior [58,59,60] As lame cows tend to have pain and be lower in rank [61], this results in different behaviors such as longer duration spend at the resting, shorter duration at feeding places or grazing [53,62]. Detection of abnormal behavior is out of the scope of this review, but a detailed overview of the link between lameness and changes in lying/standing behavior, activity, feeding behavior, behavior around milking, estrus and social behavior can be found in Van Nuffel [17].

### 4.5. Some Considerations

Several gait and behavioral characteristics change when cows develop lameness making them interesting characteristics to be used by automatic lameness detection systems. To implement them, automating the assessment of these characteristics is a prerequisite. Direct characteristics of gait (e.g., stride length) might show more sensitivity in detecting lameness compared with more indirect characteristics such as lying and feeding behavior. Indeed, the intrinsic problem with using indirect measures of lameness is that they are also influenced by other factors, thus inhibiting straightforward conclusions of causal effects. Lying behavior for example is also associated with stage of lactation, production level, lying surface, bedding, cow comfort, stocking density, stall size, stall configuration, pen lay out, milking and feeding management, social rank, *etc*. [63]. A number of these factors might also influence the ‘normal’ gait of cows as recently investigated by Van Nuffel [17]. Also, it is not clear what the cause and effect relationship is between lameness and behavior; lameness may alter behavior, or may be the consequence of the behavior [64]?

## 5. Visual Locomotion Scoring

To reduce lameness, farmers need to be aware of the number of lame cows and the severity of lameness in their herd. The commonly accepted methodologies to quantify lameness rely on spotting changes in gait, posture or behavior of the cows. In practice, this is done using subjective methods such as visual observations leading to locomotion scorings by the farmer, an employee, a veterinarian or an agricultural consultant. Subjective scoring is quick to apply, inexpensive and easy-to-use. As many as 25 different visual scoring systems are available. Most of them are based on assessing walking cows, but they differ with respect to the scale used, the gait characteristics and postures considered and the definition of lameness applied [65]. The used scales range from binary (lame *versus* non-lame) to continuous (1 to 100). On a 5-point scale from 1 to 5, cows with a score > 3 are typically defined as being clinically lame. A range of indicators, defined as specific characteristics of gait or posture relevant for lameness, are used to help the observer assess the quality of a cow’s gait and posture and score their gait in classes ranging from non-lame to severely lame.

### 5.1. Visual Locomotion Scoring Systems and Accompanying Lameness Indicators

Manson and Leaver [66] were the first to describe locomotion scoring in cattle in detail. Cows were scored using a 9-point scale based on the absence or presence of tenderness, abduction and difficulty in turning/rising/walking. Wells *et al*. [67] proposed another system mainly focusing on gait asymmetry and restriction of movement. In this system, only 5 different locomotion classes were used. Sprecher *et al*. [46] introduced a 5-point lameness scoring system that assessed gait with special emphasis on back posture, both while standing and walking. In addition, short striding and weight bearing between different limbs were used during scoring. Winckler and Willen [68] modified the Sprecher method and introduced their 5-point scoring systems using the following criteria: irregular gait, short striding and reluctance to bear weight. Breuer *et al*. [69] introduced head bobs in a 4-point scoring system. Flower and Weary [70] proposed head bobs, tracking up and joint flexion as gait indicators to look for lameness. Arc of the foot flight, foot placement relative to body position, limb axis and foot rotation during weight bearing of every limb were looked at by Dyer *et al*. [71] in their aim to identify lame and sound limbs. The Welfare Quality assessment protocol for lameness in cattle focuses on irregular footfall, uneven temporal rhythm between hoof beats and weight not borne for equal time on each of the four feet [72]. Using the presence of nine gait indicators (tenderness, stiff joints, speed, arching of the back, head bobs, irregularity in hoof placement or timing, step overlap and abduction) Van Nuffel *et al*. [73] divided cows into non-lame (no indicator present), mildly lame (1 indicator present) or severely lame (2 or more indicators present or 1 present in severe grade). Lameness assessment of cows in tie-stalls was developed by Leach *et al*. [74]. Tied cows were considered lame when two of the following indicators were visually present: repeated weight-shifting between feet, rotation of feet from the line parallel to the midline of the body, standing on the edge of a step, resting a foot, and uneven weight bearing when moving from side to side. 

In contrast to the visual locomotion scoring systems described above, some systems are based on scoring different gait characteristics separately from 1 (normal) to 5 (severely abnormal), such as tracking, spine curvature, speed, head bobbing, general symmetry and abduction/adduction [75,76,77,78].

Most of the visual locomotion scoring systems described in the literature use a specific number of classes ranging from non-lame to severely lame, often referred to as a numerical rating system (NRS). The number of classes range from 2 (lame/none lame) to 9 and allocation to a class depends on the absence or presence of gait characteristics, which differ in degrees of severity between each of these classes. Another approach uses an overall visual analogue scale (VAS). This is generally a continuous 100-unit line with at both ends of the scale the most extreme conditions of the characteristic. If VAS is used for general lameness scoring, those extremes would be ‘perfect gait’ and ‘cow unable to move’. Flower and Weary [70] suggested that such a scoring system might be more sensitive than NRS as it allows observers to record more subtle changes in gait characteristics.

Schlageter-Tello *et al*. [65] determined that the most commonly used visual scoring method is the one proposed by Sprecher *et al*. [46], which uses an ordinal scale from 1 to 5 that includes back arching as the most important characteristic to assess.

### 5.2. Practical Settings 

In lameness research there is not only a wide variety in visual locomotion scoring systems, but also in the practical settings in which that scoring is performed. This makes it even harder to compare results between different studies. Most of the publications do not describe the specific practical settings or conditions under which locomotion was assessed, although the majority can have a great impact on the scoring. In Table 2 some discrepancies between important practical settings for locomotion scoring are illustrated by means of publications that included this information.

**Table 2:** Overview of practical settings used during visual locomotion scoring.

Practical Setting	Description Used (Reference)	References
Timing of scoring	Before milking	O’Driscoll *et al*. [77]
	After milking	O’ Callaghan *et al*. [79]
Walking surface	Solid or slatted concrete	O’ Callaghan *et al*. [79]
	Rubber floor	O’Driscoll *et al*. [78]
	Wet or dry surface	
	Surface covered with slurry cleaned with automatic scrapers between every measurements	Flower and Weary [70]
Walking distance	Unspecified length	
	Specified length of e.g., 8.71 m, 7.05 m or a minimum of 10 m	Flower and Weary [70]; O’Driscoll *et al*. [78]; Wells *et al*. [67] resp.
Walking direction	Standing	Dyer *et al*. [71]
	Walking in a straight line	
	Walk into a left and right circle	
Observer angle	Walking away from the observer	
	Side view	O’Driscoll *et al*. [78]

Some of the practical settings summarized in Table 2 influence the gait of cows. For example, the amount and type of moisture on flooring surfaces influences the gait of cows because cows tend to walk more carefully (increased number of strides and decreased speed) on slippery floors compared with dry concrete floors) [80,81]. The presence of rubber and slatted flooring also influences the gait of cows [39,82]. In addition, floor roughness affects the gait of cows resulting in ‘stiffer and less confident’ walks on slippery surfaces, *i.e.*, quicker with shorter steps and less range of motion [83], compared with longer swing phases combined with long strides on surfaces with higher friction [84]. Rushen and De Passillé [81] suggested that increasing the compressibility of the walk surfaces can improve cow locomotion independent of the roughness of the surface. Flower *et al*. [16] showed that cows walk faster, with longer strides, higher stride heights, and shorter stride duration after milking compared with before milking. This might either be due to their high motivation to go back to the barn or pasture for feeding or the decreased udder distension after milking. According to Telezhenko [24] quite some variability (12.1% to 76.6%) was present within cows for locomotion variables measured during 4 consecutive strides of cows walking in a straight line, showing the difficulty of achieving stability in the locomotion indicators within the 4 different strides of one test run during this experimental set-up. These findings suggest that locomotion scores using obvious observer presence should be based on several complete gait cycles in which cows can proceed undisturbed. The influence of angle of observation or walking pattern on lameness scoring has not yet been investigated. This overview illustrates the need for practical guidelines when scoring cow locomotion both in scientific studies as in practice in order to limit the effect of variable conditions on scoring.

### 5.3. Inconsistency in Scoring

Locomotion scoring requires the observer to distinguish normal from abnormal walking behavior. Since scoring is based on observer judgment it is open to some degree of interpretation. Hence, observers should be trained and retrained by observers familiar with the scoring system in order to obtain a high degree of agreement between and within observers [85,86,87]. As with every new observation, observers gradually build up more experience with the scoring system and with the range in which indicators can be shown, they will also drift in interpretation of the borders of each specific class. Periodical re-training is therefore advised to reach an acceptable level of inter-observer reliability [88]. Using fewer locomotion classes is sometimes suggested to improve intra- and inter-observer reliability [89]. The intra- and inter-observer variation of locomotion scoring systems for cattle have been assessed in several studies [46,67,68,70,79,89] and recently been reviewed by Schlageter-Tello *et al*. [65]. Engel *et al*. [90] pointed out that when using discrete scores, cows that were in between categories might be scored in different classes by less trained and trained observers even if they had more or less the same opinion. Schlageter-Tello *et al*. [65] suggest that it is easier to score consistently with 2- or 3-scale systems compared to a 5-scale system. Thomsen *et al*. [91] found that training had only a limited positive effect on the intra- and inter-observer reliability. Using a continuous scale from 0 to 100 with extremities on both ends might reduce the variability between observers. However, a larger variation between observers was found when using a VAS compared with a numeric scale with 9 categories [90]. In the study of O’Callaghan *et al*. [79] the intra- and inter- observer reliability using a 5-point scale were 56 % and 37%. These scores increased to 93% and 81%, respectively, when a one-point difference was allowed. Tuyttens *et al*. [92] introduced a modified VAS with additional anchor points dividing the continuous scale into three equal parts, resembling ordinal scores of NRS with 3 classes. The correlation between the means of the continuous scores and the categorical scores was high (r = 0.93). This suggests that both systems can be used to provide similar information on lameness in cattle. The inter-observer reliability was higher for VAS compared with NRS. Moreover, the majority of observers (66%) preferred VAS over NRS. 

**Table 3:** Commonly used gait indicators and their most detailed definition found in literature (resp. sources of the definitions are visualized with *).

Indicator	Score	Definitions	Scientific Sources
Back arch	1	The cow stands and walks with a level-back posture	Sprecher *et al*. [46] *; Flower and Weary [70]; Dyer *et al*. [71]; Gleeson *et al*. [75]; Olmos *et al.* [76].
	2	The cow stands with a level back posture but develops an arched-back posture while walking	
	3	An arched-back posture is evident both while standing and while walking	
	4	An arched-back posture is always evident	
	5	The cow additionally demonstrates inability or extreme reluctance to move	
Head bob	1	Normal vertical movement during locomotion	Whay *et al.* [97]; Flower and Weary [70]; Gleeson *et al.* [75] *; Olmos *et al.* [76].
	2	Head ‘nods’ during locomotion	
	3	Marked vertical head movement during locomotion	
	4	Severe vertical movement during locomotion	
	5	Head lowered almost to ground level with each step	
Tracking up	1	Hind footprint fully traces, or is more forward than front footprint	Whay *et al.* [97]; Olmos *et al.* [76] *; Flower and Weary [70]; Gleeson *et al.* [75]; Olmos *et al*. [76].
	2	Hind footprint partly traces (slight behind) front footprint	
	3	Toe or hind footprint reaches heel of front footprint	
	4	Hind footprint approx. 30 cm behind front footprint	
	5	Hind footprint more than 30 cm behind front footprint	
Joint flexion	0	Flexes and extends limbs through the normal range of motion;	Wells *et al.* [67]; Winckler and Willen [68]; Flower and Weary [70] *.
	100	Limited flexion and extension resulting in stiffness	
Tenderness		No clear definition found	Manson and Leaver [ 66]; Winckler and Willen [68]; Flower and Weary [70]; Dyer *et al.* [71].
Asymmetric steps	0	Gait abnormality not visible during walk	Wells *et al.* [67] *; Manson and Leaver [66]; Winckler and Willen [68]; Flower and Weary [70]; Olmos *et al.* [76]
	1	Mild variation from normal walk; includes intermittent gait asymmetry or mild bilateral or quadrilateral restriction of free movement	
	2	Moderate or consistent gait asymmetry or symmetric gait abnormality	
	3	Marked gait asymmetry or severe symmetric abnormality	
	4	Recumbent	
Reluctance to bear weight or Equal distribution weight	1	Normal gait	Winckler & Willen [68] *; Flower and Weary [70]; Dyer *et al.* [71]
	2	Uneven gait (stiff/very careful/swinging of legs around the udder/swaying of trunk and hind quarters)	
	3	Short striding gait with one limb (even if just noticeable)	
	4	Short striding gait with more than one limb or strong reluctance to bear weight on one limb	
	5	Does not support on limb in one or more limbs, holding a limb up whenever possible.	
Ease of movement/turning	1	Smooth and fluid movement	Manson and Leaver [66]; Wells *et al.* [67]; Dyer *et al.* [71]; Olmos *et al.* [76] *
	2	Ability to move freely is diminished	
	3	Capable of locomotion but ability to move freely is compromised	
	4	Ability to move freely is obviously compromised	
	5	Ability to move is severely restricted. Cow must be vigorously encourage to stand or move	
Abduction/adduction	1	Hind limb moves forward parallel to vertical midline of animal	Manson and Leaver [66]; Gleeson *et al.* [75] *; Olmos *et al.* [76].
	2	Slight deviation from midline of animal	
	3	Hooves form a C shape in the air as they move forward	
	4	C shape so defined as to be almost circular	
	5	Hooves circle completely in the air between each step	
Stride or step length	1	Normal gait	Sprecher *et al.* [46]; Winckler and Willen [68] *; Dyer *et al.* [71].
	2	Uneven gait (stiff/very careful/swinging of legs around the udder/swaying of trunk and hind quarters)	
	3	Short striding gait with one limb (even if just noticeable)	
	4	Short striding gait with more than one limb or strong reluctance to bear weight on one limb	
	5	Does not support on limb in one or more limbs, holding a limb up whenever possible.	
Speed	1	Normal locomotion at a comfortable pace	Dyer *et al.* [71]; Gleeson *et al.* [75]; Olmos *et al.* [76]; O’Driscoll *et al.* [77] *
	2	Locomotion slower than normal	
	3	Slow, slightly hesitant walk and/or slight reluctance to bear weight	
	4	Very slow hesitant walk and/or reluctance to bear weight	
	5	Cow unable/unwilling to walk	

Channon *et al*. [89] suggested that the root of the reliability problem could be the qualitative nature of the scoring systems, which are rather vague and indistinct. Without clear and objective descriptions, a scoring method is open to individual interpretation [93]. For example, using classes including ‘moderately lame’ and ‘slightly lame’ means that unless observers have viewed a large number of cows and developed agreed classes, there is no basis for reliable scoring. Moreover, the fact that ‘lame’ as a gait indicator is used as the basis for a lameness score is rather confusing. However, some scoring systems give a more thorough description of the indicators used to define the locomotion score. A summary of the most commonly used gait indicators in visual locomotion scoring systems is provided in Table 3. For every gait indicator, the most detailed and clear description found in the literature is given, together with other scientific papers using this indicator. Despite the efforts to clearly define the different levels of gait indicators, many scoring systems rely on only one or a few specific indicators, such as arched back. This might disguise lameness problems compared with scoring systems that use a wider range of indicators [93].

Another problem is the lack of good scientific descriptions on how healthy cows walk. The indistinct description of ‘normal gait’ or the ‘gait of non-lame cows’ in Table 4 implies that scoring cow gait objectively and reliably can only be possible with well-defined criteria and classes and with extensive experience in locomotion scoring to have a wide range of reference gaits to compare with. To define reliable and repeatable scoring systems, improved knowledge of the gait of cows is thus required. This can be derived from computer-assisted kinematic techniques that obtain precise measures of gait and how this gait changes when lameness occurs (von Keyserlingk *et al*., 2009) [93]. Such kinematic gait analyses are usually restricted to gait laboratories and are difficult to introduce into commercial barns due to the use of body markers or their specific needs for lighting. Nevertheless, the interest in improved and automated methods for gait monitoring increases.

**Table 4:** Overview of criteria for non-lame cows found in frequently used scoring systems.

Source	Description for Non-Lame Cows in Scoring System
Manson and Leaver [66]	Minimal abduction/adduction, no unevenness of gait, no tenderness
Wells *et al*. [67]	Gait abnormality not visible at a walk; not reluctant to walk
Sprecher *et al.* [46]	Stands and walks with a level-back posture. The gait is normal
Breuer *et al.* [69]	Normal gait
Winkler and Willen [68]	Normal gait
Flower and Weary [70]	Smooth and fluid movement with flat back, steady head carriage, hind hooves land on or in front of fore-hooves (track up), joints flex freely, symmetrical gait and all legs bear weight equally
Dyer *et al.* [71]	Cows walk freely with unrestricted motion and symmetry in stride. No postural abnormalities are exhibited.
Welfare Quality^®^ [72]	Timing of steps and weight-bearing equal on all four feet

### 5.4. Practical Considerations 

Although visual locomotion scoring can be implemented on any farm at any given time, some practical considerations should be taken into account. Besides the inconsistency of the locomotion scoring, assessing lameness of individual cows in a herd can be practically challenging. Although probably possible to construct, there is generally no specially designed alley available on farms where cows pass daily while their gait is observed. Cows often have to be assessed in barns lacking sufficient space to walk in a straight line for several meters. The slatted floor is furthermore mostly covered with slippery manure impairing normal locomotion. Also, cows can be reluctant to walk past herd mates and need to be motivated to walk past them. This again disturbs their normal locomotion and increases the need for an additional helper to guide the cow in order for the observer to give a score. Being a prey species and hence stoic in nature, lame cows might also mask signs of vulnerability, because this would make them an easier target for predators [94]. Therefore, in the presence of an observer or when forced to walk, cows tend to only show lame locomotion and behavior when the lameness is at an advanced stage. As only a trained observer may notice the onset of lameness by multiple subtle gait aberrations, detecting early lameness tends to be difficult. In addition, as discussed above, practical guidelines on the set-up for visual locomotion scoring are lacking. 

Most visual scoring systems are not designed to assess lameness on a day-to-day basis, but to compare locomotion scores between different treatments or set-ups. Sporadic scoring of the entire herd by an experienced observer only provides the farmer or veterinarian with prevalence information for that specific moment. Because of the non-straightforward link between lameness and claw lesions or other diseases of the bones and joints [95], the usefulness of locomotion scores is limited to indications of pain, rather than as a diagnostic tool for the presence of hoof lesions [68]. This makes a thorough check of the cow necessary. 

More importantly, all cows have their own specific way of walking [17] and reacting on illness [96]. Hence, a detection system should rather be based on changes in indicators over time than on the deviation from the group mean. Daily monitoring of locomotion or behavior on farm by a trained observer is too time-consuming and thus very cost inefficient. Automatic measurement of lameness-related, animal-based characteristics would allow for daily measurements and could, therefore, be a better option.

## 6. Conclusions 

Clear and accurate definitions including numerical information of normal and abnormal cow gait are lacking in the description of visual locomotion scoring system. This adds to the difficulty of performing visual locomotion scoring to assess lameness on farms. Moreover, the farm design, experimental set-ups and presence of an observer impede proper scoring on practical farms even more. Different practical approaches are used during locomotion scoring, resulting in incomparable results between studies. The different set-ups used and their influence on the resulting locomotion score was evaluated. Automated measurements of lameness-related cow features might therefore solve some of these issues. However, the practical set-up of such systems will also be challenging.
